# Predictors of Shoulder Pain and Disability Index (SPADI) and work status after 1 year in patients with subacromial shoulder pain

**DOI:** 10.1186/1471-2474-11-218

**Published:** 2010-09-23

**Authors:** Kaia Engebretsen, Margreth Grotle, Erik Bautz-Holter, Ole Marius Ekeberg, Jens Ivar Brox

**Affiliations:** 1 Department of Physical Medicine and Rehabilitation, Oslo University Hospital, Ullevaal, and Medical Faculty, University of Oslo, Norway; 2 FORMI, Division for Neuroscience and Musculoskeletal Medicine, Oslo University Hospital, Ullevaal, Norway; 3 National Resource Centre for Rehabilitation in Rheumatology, Diakonhjemmet Hospital, Oslo, Norway; 4 Department of Orthopaedics, Section for Physical Medicine and Rehabilitation, Oslo University Hospital, National Hospital, and Medical Faculty, University of Oslo, Norway

## Abstract

**Background:**

Shoulder pain is a common complaint in primary health care and has an unfavourable outcome in many patients. The objectives were to identify predictors for pain and disability (SPADI) and work status in patients with subacromial shoulder pain.

**Methods:**

Secondary analyses of data from a randomized clinical controlled trial were performed. Outcome measures were the absolute values of the combined Shoulder Pain and Disability Index (SPADI) and work status 1 year after treatment with supervised exercises (SE) or radial extracorporeal shockwave therapy (rESWT). Predictors of outcome were investigated using multiple linear regression (SPADI) and logistic regression (work status).

**Results:**

104 patients were included. Low education (≤ 12 years), previous shoulder pain, and a high baseline SPADI score predicted poor results with these variables explaining 29.9% of the variance in SPADI score at 1 year. Low education and poor self-reported health status predicted a work status of "not working": Odds Ratio, OR = 4.3(95% CI (1.3 to 14.9)), p = 0.02 for education, and OR = 1.06 (95% CI (1.0 to 1.1)), p = 0.001 for self-reported health status, respectively. Adjustments for age, gender, and treatment group were performed, but did not change the results.

**Conclusion:**

Education was the most consistent predictor of pain and disability, and work status at 1 year follow-up. Also, baseline SPADI score, previous shoulder pain and self-reported health status predicted outcome.

**Trial registration:**

Clinical trials NCT00653081

## Background

Shoulder pain is a common complaint in primary health care with a 1-year prevalence ranging up to 47% in the adult population [1-3]. Rotator cuff disease, rotator cuff tendinosis, and impingement syndrome are terms used synonymously with subacromial shoulder pain. The pain condition has an unfavourable outcome in many patients and may impose a burden on the individual and society [2,4,5].

In general, most patients with musculoskeletal pain are pain-free within a few weeks [6]. However, some patients develop chronic pain. To indentify the influencing factors or predictors might be important for outcome [7].

Prognosis of shoulder pain may be influenced by different factors or a combination of factors such as socio-demographics, genetics, psychological- and personal-traits, occupational factors, work status, characteristics of the shoulder pain, use of medication, and treatment [8,9]. According to a systematic review, few high quality prognostic studies exist [10]. There is some evidence that high pain intensity predicts poorer outcome in primary care populations and that middle age is associated with poor outcome in occupational populations [8,11].

Better knowledge about predictors of outcome may help to identify patients with good prognosis and patients at risk for long term disability. In addition, this may be helpful in improving design and analysis in research. It is suggested that health resources may be better allocated if psychosocial factors related to work absence are identified [12].

We have previously reported that while supervised exercises (SE) or arthroscopic surgery improved the prognosis in patients with rotator cuff tendinosis or subacromial pain, sick leave and regular medication were negatively associated with outcome [13,14]. Self-reported shoulder related work-demands (physical and psycho-social) were not associated with outcome for this patient group, while other studies have reported that physically demanding factors related to physical work may be of importance [8,10].

The objectives of the present study were to identify predictors for pain and disability (SPADI) and work status 1 year after non-operative treatment in patients with subacromial shoulder pain.

## Methods

### Study population

The study population was recruited by physicians at the outpatient Department of the Physical Medicine and Rehabilitation at Oslo University Hospital, Ullevaal, Norway between July 2006 and August 2007. They were included in a clinical randomized study comparing supervised exercises (SE) with radial extracorporeal shockwave therapy (rESWT) [15,16]. The patients were between 18 and 70 years old and had had subacromial shoulder pain for at least 3 months. The inclusion criteria were: dysfunction or pain on abduction; a normal passive glenohumeral range of motion; pain on two of three isometric tests (abduction at 0° or 30°, external or internal rotation); and a positive impingement sign. The exclusion criteria were: bilateral shoulder pain, previous surgery on the affected shoulder, instability, referred pain from neck, rheumatoid arthritis, clinical and radiological signs of glenohumeral- or acromioclavicular arthritis, serious somatic or psychiatric disorder or inability to understand Norwegian.

Patients gave their informed signed consent after written and verbal information before baseline registration. One hundred and four patients were included (50% women). Both treatments were conducted at the outpatient Department of Physical Medicine and Rehabilitation Ullevaal, Oslo University Hospital. The supervised exercise regimen (SE) was provided by two physiotherapists and the patients attended two 45-minute sessions weekly for a maximum of 12 weeks [15]. Radial extracorporeal shockwave therapy (rESWT) (Swiss Dolor Clast, EMS) was provided by another physiotherapist, and administered once a week for 4 to 6 weeks, with 3 to 5 tender points treated each time [15].

The study protocol was approved by the Ethics Committee for Medical Research, Health region I, Norway.

### Potential predictors and outcome measures

At baseline, potential predictors previously identified in prospective and rehabilitation studies were assessed [8,11,17,18]. Socio-demographic variables included age, gender, educational level (≤12 years at school) and work status (working >50%). Status of "not working" included those on sick leave, disability pension and vocational rehabilitation. Status of "retired" was not included. Characteristics of shoulder complaints included intensity and duration of pain (3-6 months, 6-12 months, >12 months), previous shoulder pain, previous treatment, and dominant arm involvement. Occupational factors examined were frequency of heavy lifting and working above shoulder level, which were classified into 3 categories (seldom/never, sometimes, extremely/often) [19]. Use of pain medication, sleeping medication, and relaxation medication was registered according to frequency of use (not regularly versus daily or weekly). Emotional distress was scored from one to four by the 25-items Hopkins Symptoms Checklist with a higher score indicating more distress [20]. General health status was evaluated by EQ-VAS (0-100) with higher scores indicating better perceived health [21]. Self-efficacy for pain was evaluated as the sum of 4 items from 1(easy) to 7 (impossible) [22]. Active range of motion (AROM) including hand-behind-back (HBB: the position of the thumb in reference to the pelvic (trochanter major = 1)) was also assessed [23,24].

The Shoulder Pain and Disability Index (SPADI) at 1 year follow-up was used as the primary outcome [25,26]. We used the absolute values of the combined score which consists of 5 pain and 8 disability items and measures pain and disability for both current status (last week) and change over time [27]. It is rated on horizontal visual analogue scales (VAS) that range from 0 to 11[27]. The scores are added and form a total score ranging from 0 to 100 points where a higher score indicates more shoulder pain and disability [27].

Work status at 1 year was used as the secondary outcome. It was included as a secondary outcome variable because of its importance to both the patients and the society.

### Statistical analysis

Two separate univariate regression analyses were performed to examine the relationship between each of the putative predictors and the two outcome measures at 1 year follow-up.

Variables were examined for linear relationships and the correlation had to be less than 0.7 for the predictor to be included in the univariate analysis [28]. Predictors that were associated with the outcome with p < 0.1 were included in a multiple linear regression model (SPADI). Manual backward elimination was performed. Age, gender and treatment group were kept in the model (adjustment) and variables with p ≥ 0.05 were removed manually. The percentage of explained variance (R^2^) was calculated to give an indication of the predictive power of the final multiple regression model.

Logistic regression was applied to predict work status. Forward selection was used because only 10% of the lowest category (i.e. the analysis required 10 people not working at 1 year for each predictor entered in the model) of work status at 1 year was allowed simultaneously in the mathematical model.

In the final model for pain and disability all possible interactions (between the independent variables) were evaluated.

The Hosmer and Lemeshow Test (p > 0.05) assessed the "goodness of fit" of the logistic model, and R Square tests (Cox&Snell and Negelkerke) provided an indication of the amount of variation (min 0, max approximately 1) [29]. The area under the receiver-operating characteristic curve (ROC) was used in order to assess the discriminative ability of the model whereas the true positive rate (sensitivity) was plotted against the false positive rate (1-specifity). An area under the curve (AUC) of 0.5 indicates no discrimination above chance, whereas an AUC of 1.0 indicates perfect discrimination.

## Results

### Study population

**Figure 1:**
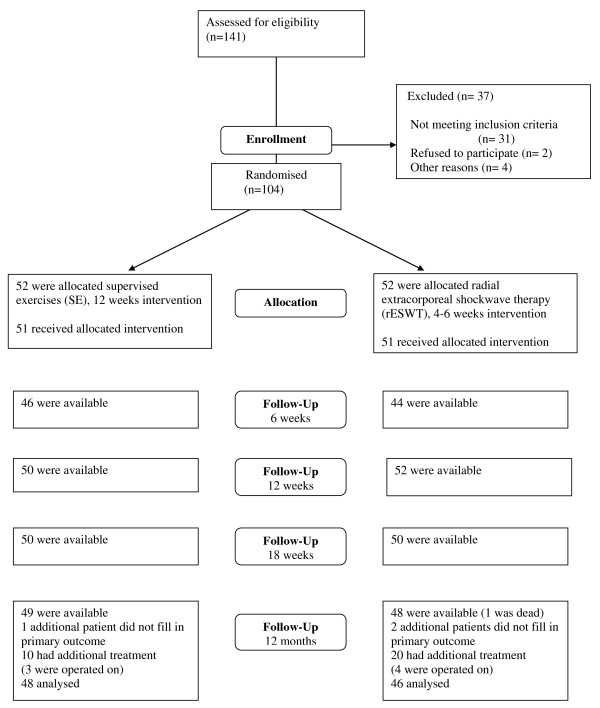
**Flow chart of the study**.

Baseline characteristics of the 104 patients are shown in table 1. Of the included patients, 90% completed and returned the questionnaire, 94 patients completed SPADI and 91 the questions regarding work status (fig 1).

**Table 1:** Baseline characteristics of the patient population (n = 104)

	Frequency	Mean
	(percent)	(SD)
**Socio-demographic variables:**		
Age		48 (10.7)
Education:		
≤ 12 years at school	60 (57.7%)	
University/College	44 (42.3%)	
Work status:		
Working	57 (54.8%)	
Not working	41 (39.4%)	
Retired	6 (5.8%)	
**Variables from questionnaire:**		
Duration of pain:		
3-6 months	34 (32.7%)	
6-12 months	30 (28.8%)	
>12 months	40 (38.5%)	
Medication:		
Daily or weekly	49 (47.1%)	
Not regularly	55 (52.3%)	
Scorings:		
SPADI*		46.9 (21.3)
Pain at rest (9-point)		3.5 (2.0)
Health status (EQ-VAS)		67.7 (18.3)
Prev shoulder pain (0,1)	64 (61.5%)	
Previous physiotherapy (0,1)	47 (45.2%)	
Distress (HSCL 25)		1.5 (0.47)
Self-efficacy for pain 1(easy- impossible)		3.9 (1.3)
Neck pain	48 (46.6%)	
Occupational factors:		
How often carry 10 kilos at work?		
seldom/never	50 (53.8%)	
sometimes	35 (37.6%)	
extremely/often	8 (8.6%)	
How often work above shoulder level?		
seldom/never	36 (38.7%)	
sometimes	38 (40.9%)	
extremely/often	19 (20.4%)	
**Active range of motions affected side:**		
Flexion aff side		157.6 (25.1)
Hand- behind- back**		13 (median)

Continuous variables: Mean values ± SD, Categorical variables: Frequency, median, %

* The Shoulder Pain and Disability Index

Self-efficacy for pain: A sumscore of 4 questions (mean)

**The position of the thumb in reference to the pelvic (trochanter major = 1) and level of columna

Eight patients who did not answer the 1 year follow-up were not working at baseline. The missing subjects at the 1 year follow-up were older (57 years versus 49 years), and had a higher mean baseline SPADI score (56 versus 49) compared with the whole study group.

### Predictors of pain and disability at 12 months

Table 2 presents the univariate association of potential predictors of pain and disability at the 1 year follow-up. Pain on activity and the two questions related to function were taken out because of high inter-correlation (r > 0.7). The variables; "how often do you carry 10 kg at work" and "how often do you work above shoulder level", were not significantly associated with outcome (0.11< p < 0.91). Education, work status, distress, EQ-VAS, pain at rest, previous shoulder pain, baseline SPADI score, self-efficacy for pain, flexion, and hand-behind-back (HBB) were included in the multiple regression analyses. Low education, previous shoulder pain, and high baseline SPADI score predicted higher SPADI scores (more pain and disability) after 1 year. The variables in the adjusted final model for predicting pain and disability (high SPADI scores) are presented in table 3. The model predicted 29.9% (R^2^) of the variance.

**Table 2:** Univariate linear regression for the dependent variable SPADI after 1 year and coefficients with CI for Beta

SPADI total				
	R^2 ^	Beta	95% CI (for B)	p-value
n = 104				
**Socio-demographic variables:**				
Gender (1,2)	0.7%	4.05	(-6.2 to 14.3)	p = 0.44
Age	0.9%	-0.24	(-0.75 to 0.27)	p = 0.35
Education (0,1)	14.4%	-18.9	(-28.4 to -9.4)	p < 0.01
Work status (0,1)	8.0%	14.7	(4.1 to 25.3)	p = 0.007
**Variables from self-reported questionnaire:**				
Duration of pain (0-2)	4.5%			
3-6 months^b^				
6-12 months		4.0	(-9.1 to 17.2)	p = 0.54
> 12 months		9.7	(-0.62 to 20.1)	p = 0.065
Medication (0,1)	0.7%	4.1	(-6.2 to 14.4)	p = 0.43
Distress (HSCL 25)	3.4%	10.3	(-1 to 21.6)	p = 0.073
Health stat (EQ-VAS)	7.5%	-0.4	(-0.7 to -0.1)	p = 0.008
Pain at rest	11.9%	4.3	(1.9 to 6.7)	p = 0.001
Self-eff for pain(sum)	8.9%	6.0	(2.0 to 9.9)	p = 0.004
Baseline SPADI	14.3%	0.46	(0.22 to 0.67)	p < 0.001
Prev should pain(0,1)	5.2%	11.5	(1.3 to 21.8)	p = 0.028
Neck pain (0,1)	2.0%	7.0	(-3.3 to 17.3)	p = 0.18
Prev physiot(0,1)	2.6%	8.0	(-2.2 to 18.2)	p = 0.12
**Active range of motions affected side (impairments):**				
Flexion	3.3%	-0.18	(-0.39 to 0.21)	p = 0.078
Hand-Behind -Back	4.1%	-1.75	(-3.5 to -0.004)	p = 0.05

b Reference category

Gender (1: male, 2: female)

Education (0: ≤ 12 years in school, 1: College/University)

Work status (0: not working, 1: working or partly working, retired not included)

Medication (0: no regular use of medicine, 1: daily or weekly use of medicine)

Self-efficacy for pain: a sumscore of 4 questions (mean)

Previous shoulder pain (0: no,1: yes)

SPADI: 11 missing, Work status: 13 missing

**Table 3:** Multiple regression model (backward) adjusted for treatment group, gender and age with Beta values, 95% CI for Beta, the Shoulder Pain and Disability Index (SPADI) at 1 year as the dependent variable and the total percentage of variance (R^2^)

SPADI			
	Beta	95% CI for Beta	p-value
n = 94			
Education (0,1)	-14.3	(-23.5 to -5.2)	p = 0.003
Previous shoulder pain (0,1)	11.0	(1.4 to 20.6)	p = 0.026
Baseline SPADI	0.37	(0.15 to 0.59)	p = 0.001

Total R^2 ^     29.9%

Age, treatment group and gender were adjusted for in the model

Education (0: ≤ 12 years in school, 1: University/College)

Previous shoulder pain (0: no, 1: yes)

We found a significant interaction term between baseline SPADI * (treatment group) in the final multiple regression model. Performing separate analysis (according to treatment group) showed a Beta value for baseline SPADI of 0.66 (95% CI (0.4 to 0.9)) for those treated with SE compared to a Beta value of 0.13 (95% CI (-0.21 to 0.47)) for those treated with rESWT.

### Predictors of work absence at 12 months

Twenty-three patients (25%) were not working after 1 year. Previous physiotherapy and all variables in the multiple regression analysis except distress, pain at rest, and previous shoulder pain were significant in an univariate logistic regression analysis, table 4.

**Table 4:** Univariate logistic regression for the dependent variable work status after 1 year with Beta, Odds ratio (OR), CI for OR, and p-values

Work status			
	Beta (for OR)	OR (95% CI)	p-value
n = 104			
**Socio-demographic variables**:			
Gender (1,2)	0.35	1.4 (0.55 to 3.7)	p = 0.47
Age	-0.001	1.0 (0.95 to 1.1)	p = 0.96
Education (0,1)	1.4	4.1(1.4 to 12.2)	p = 0.013
Work status (0,1)	1.25	3.5 (1.3 to 9.3)	p = 0.013
**Variables from self-reported questionnaire:**			
Duration of pain (0-2)			
3-6 months^b^			
6-12 months	-0.76	0.47 (0.13 to 1.7)	p = 0.24
> 12 months	-0.6	0.54(0.16 to 1.8)	p = 0.32
Medication (0,1)	-0.32	0.72 (0.3 to 1.9)	p = 0.5
Distress (HSCL 25)	-0.45	0.64 (0.24 to 1.7)	p = 0.38
Health stat (EQ-VAS)	0.056	1.06 (1.03 to 1.1)	p < 0.001
Pain at rest	-0.17	0.84(0.66 to 1.07)	p = 0.15
Self-eff for pain(sum)	-0.4	0.67(0.5 to 1.0)	p = 0.052
Baseline SPADI	-0.028	0.97(0.95 to 1.0)	p = 0.025
Prev should pain(0,1)	-0.9	0.42 (0.15 to 1.2)	p = 0.1
Neck pain (0,1)	-0.62	0.54 (0.21 to 1.4)	p = 0.2
Prev physiot(0,1)	0.86	2.3 (0.9 to 6.2)	p = 0.082
**Active range of motions affected side (impairments):**			
Flexion	0.017	1.02 (1.0 to 1.04)	p = 0.067
Hand-Behind -Back	0.15	1.2 (0.99 to 1.4)	p = 0.064

b Reference category

Gender (1: male, 2: female)

Education (0: ≤ 12 years in school, 1: College/University)

Work status (0: not working, 1: working or partly working, retired not included)

Medication (0: no regular use of medicine, 1: daily or weekly use of medicine)

Self-efficacy for pain: a sumscore of 4 questions (mean)

Previous shoulder pain (0: no,1:yes)

SPADI: 11 missing, Work status: 13 missing

The variables in the adjusted final model for predicting work status are presented in table 5. Higher education and also better self-reported health status predicted working after 1 year: OR of 4.3 (95% CI (1.3 to 14.9)), p = 0.02 for education, and OR = 1.06 (95% CI (1.0 to 1.1)), p = 0.001 for reported health status, respectively. The odds of working are 4.3 times higher for those with more than 12 years of education. A one point higher score on EQ-VAS increase the probability of working with 6%. Seventy-three percent of those working were located in the 2 upper percentiles (scores of 70-100) compared to 17% of those who were not working.

**Table 5:** Logistic regression model (forward) with Beta values, Odds ratios (OR), 95% confidence intervals (CI) for OR, work status at 1 year as the dependent variable

WORK STATUS				
	Beta	OR	95% CI for OR	p-value
n = 90				
Education (0,1)	1.5	4.3	(1.3 to 14.9)	p = 0.02
Health status (EQ-VAS)	0.06	1.06	(1.0 to 1.1)	p = 0.001

Age, treatment group and gender were adjusted for subsequently

Work status (0: not working, 1: working or partly working, retired not included)

Education (0: ≤ 12 years in school, 1: University/College)

Adjusting subsequently for gender, age and treatment group did not change this model. The reliability of the model was acceptable according to the Hosmer-Lemeshow statistic with p = 0.46, and R^2 ^between 22% and 33%.

The AUC for the final logistic regression model at 1 year was 0.78 (95% CI (0.67 to 0.9)).

## Discussion

Education was the most consistent predictor of a poor outcome of pain and disability (SPADI) and work status at 1 year follow-up. In agreement with previous studies also self-reported health status, previous shoulder pain, and pain and disability (higher SPADI score) at baseline, predicted poor outcome [10,17,30].

All patients satisfied clinical criteria for subacromial pain and were included in a clinical trial [15]. Strictly, our findings are valid for patients fulfilling the criteria outlined. We did not include a placebo group and can not estimate the prognosis as compared with patients given no treatment. However, a previous study reported that patients fulfilling similar clinical criteria randomized either to surgery or supervised exercises had better prognosis than patients given placebo laser [13].

Lower education and pain and disability (SPADI) at baseline was significant predictors for pain and disability (SPADI) after 1 year [14,31]. According to a previous study [16], patients with severe symptoms were more likely to receive more extensive treatment, but this did not improve outcome [16,32]. Further investigation is needed to determine whether early intervention, especially for those with low education, positively affects outcome.

Work status at baseline was significantly associated with SPADI and working in the univariate analyses, but not in the multivariate analyses. The odds ratio for not working at 1 year was four-fold higher in patients with low versus patients with high education. This is well known according to back pain, but also seems to be applicable to patients with chronic subacromial pain [33]. Education is often considered to be the best indicator of socioeconomic status [34]. High educational attainment is also related to better personal economy, socio-psychological resources, and a healthy lifestyle [17,31,33]. We found that also high score on EQ-VAS, evaluating current health status predicted working after 1 year. Lower health status may affect work status because both work-related and individual factors are associated with sick leave and might influence the results [35,36]. However, the reasons for not working may also depend on the physician, as well as individual attitudes towards being sick-listed [35]. Some kind of work is difficult to perform with a painful shoulder, but previous studies suggest that long term sick-listing negatively influence return to work [13,14,36]. In agreement with a previous study from our hospital [13,14] self-reported physical work related factors evaluated were not independently associated with outcome. Few studies have included psychosocial factors in their analyses [10].

A combination of predictors seems more important than single predictors [37,38]. This supports the consensus of musculoskeletal pain as being a multidimensional problem [37]. Although many of these predictors are not in the control of health providers, such knowledge may improve the treatment decision process [9].

Separate analysis of the interaction term (baseline SPADI * (treatment group)) indicate that baseline SPADI score is a more important predictor for pain and disability after 1 year for the supervised exercise (SE) group than for those treated with rESWT. Significant interactions are of clinical interest because different subgroups may respond differently according to the outcome. Further investigation with sufficient sample size is needed.

### Advantages and limitations

Advantages of the present study are the use of the recommended clinical diagnostic criteria for subacromial shoulder pain, application of recommended outcomes, the inclusion of several possible predictors in the analyses, and performance of analyses according to recommended criteria [39-42].

The limitations are the small sample size and that missing values were not imputed. In particular, 8 patients who were not working at baseline and dropped out at 1 year may bias the results for work status and lower the possibility to find work status as a significant predictor. Patients in a clinical trial may have different prognosis compared to those excluded, but we were not able to evaluate those excluded.

Numerous predictors were investigated, but the final linear model explained no more than 30% of SPADI's variance [17]. Similar results were found for the logistic model, which suggests that between 22% and 33% of the variability was explained by the two predictors. Heterogeneity of the patient group despite uniform clinical criteria, measurement error of the outcome variables, and prognostic factors not examined, may contribute to the moderate percentage of variance accounted for [16,17]. The AUC of the model of 0.78 may be interpreted as satisfactory discrimination between patients who were working and those who were not working after 1 year. It should be noted however, that a small sample size with a relatively high number of predictors investigated tends to over-fit the predictive model and spuriously overestimate associations between factors and outcome [43].

An external validation of the results ought to be performed in different populations, preferably in a population from a cohort study and in patients in primary care [17,44]. Validation studies are of importance, especially if the request is to implement a hospital (secondary) care model to the primary care [45]. Only a selective group of patients in general practice are referred to a specialist. These patients may be considered as subgroups of patients which may affect the generaliseability.

Adjusting for treatment is recommended as well as including treatment as a separate predictor in the models, especially when the treatment has effect [45]. The treatments may change the prognosis or modify the effect [46]. The two treatments applied may also be adapted according to changes in the patients' symptoms, complicating the interpretation of treatment as a predictor [11]. However, the predictive value of treatments is often small in long term follow-up studies.

Another way to study possible predictors might be to include a population with different diagnoses of shoulder pain. Such studies require a larger sample size in order to have sufficient statistical power for subanalyses of different diagnostic groups. A more explanatory approach might be required [28].

### Clinical value

When predictive models are obtained from randomized trials, data may have restricted generaliseability due to strict eligibility criteria for the trial, recruitment level, or if large numbers refused consent [45]. In the present study, relatively few eligible patients refused to participate. In addition, the study population consisted of participants working in a wide variety of occupational settings, which makes the results more generalisable than a selective sample of workers.

The models presented, although not validated, may provide adequate information about prognostic outcome in patients with subacromial pain. They are more objective than subjective impression and can be complementary to clinical intuition [47]. The models might help clinicians to make decisions, and in giving advice to the patients. In general, few models are validated in new patient groups and implemented into clinical practice [45].

Our results indicate that generic factors not related to the shoulder problem were the most important prognostic factors, and that they may be of more importance for patients with poor outcome. This is consistent with other studies in patients with musculoskeletal pain disorders [6,7,13,33,48].

## Conclusion

We conclude that 12 or fewer years of education was the most consistent predictor of a poor outcome of pain and disability (SPADI score) and work status at 1 year follow-up. Baseline SPADI score, previous shoulder pain, and poor self-reported health status also predicted outcome.

## Competing interests

The authors declare that they have no competing interests.

## Authors' contributions

JIB, MG, KE, and EB-H contributed in the planning process of the present study including the study design. KE recruited the patients. KE and OME performed the statistical analysis. JIB and MG helped to draft the manuscript. All authors read and approved the manuscript.

## Pre-publication history

The pre-publication history for this paper can be accessed here:

http://www.biomedcentral.com/1471-2474/11/218/prepub
